# COVID-19 in der geburtshilflichen Anästhesie: Prospektive Erfassung von SARS-CoV-2-Infektionen zum Zeitpunkt der Geburt sowie des peripartalen Verlaufs SARS-CoV-2-positiver Schwangerer

**DOI:** 10.1007/s00101-021-01068-6

**Published:** 2021-11-23

**Authors:** Magdalena Sitter, Tobias Schlesinger, Ann-Kristin Reinhold, Axel Scholler, Christian von Heymann, Sabine Welfle, Catharina Bartmann, Achim Wöckel, Stefan Kleinschmidt, Sven Schneider, André Gottschalk, Susanne Greve, Julius Z. Wermelt, Roland Wiener, Frank Schulz, Daniel Chappell, Maya Brunner, Claudia Neumann, Patrick Meybohm, Peter Kranke, Weitere Mitglieder des COALA-Registers, A. Brenner, A. Foer, D. Bremerich, G. Lotz, T. Girard, Y. Zausig, L. Kaufner, M.-L. Fingerhut, M. Schick, M. Wenk, S. Klaschik, W. Zink

**Affiliations:** 1 Klinik und Poliklinik für Anästhesiologie, Intensivmedizin, Notfallmedizin und Schmerztherapie, Universitätsklinikum Würzburg, Oberdürrbacher Str. 6, 97080 Würzburg, Deutschland; 2 Frauenklinik und Poliklinik, Universitätsklinikum Würzburg, Würzburg, Deutschland; 3 Anästhesiologische Klinik, Universitätsklinikum Erlangen, Erlangen, Deutschland; 4 Klinik für Anästhesie, Intensivmedizin, Notfallmedizin und Schmerztherapie, Vivantes Klinikum im Friedrichshain, Berlin, Deutschland; 5 Klinik für Anästhesiologie, Intensivmedizin und Schmerztherapie, Universitätsklinikum des Saarlandes, Homburg, Deutschland; 6 Klinik für Anästhesiologie, Intensiv‑, Notfall- und Schmerzmedizin, DIAKOVERE Friederikenstift & Henriettenstift, Hannover, Deutschland; 7 Klinik für Anästhesiologie und Intensivmedizin, Medizinische Hochschule Hannover, Hannover, Deutschland; 8 Klinik für Anästhesie und Kinderanästhesie, Bürgerhospital und Clementine Kinderhospital gGmbH, Frankfurt am Main, Deutschland; 9 Klinik für Anästhesiologie, Universitätsklinikum Heidelberg, Heidelberg, Deutschland; 10 Klinik für Anästhesiologie, Klinikum der Universität München (LMU), München, Deutschland; 11 Anästhesiologie, Universitätsspital Basel, Basel, Schweiz; 12 Klinik für Anästhesiologie und Operative Intensivmedizin, Universitätsklinikum Bonn, Bonn, Deutschland

**Keywords:** Geburtshilfliche Intensivmedizin, COVID-19-Pademie, Infektionswellen, ECMO-Therapie, Geburtshilfe, Maternal critical care, COVID-19 pandemic, Waves of infection, ECMO-therapy, Obstetrics

## Abstract

**Hintergrund:**

Im Rahmen der Pandemie des SARS-CoV-2-Virus erlangte das Patientenkollektiv der Schwangeren früh Aufmerksamkeit. Initial wurde angesichts sich früh abzeichnender Krankheitsfälle bei jüngeren Patienten mit einem erheblichen Aufkommen peripartal zu betreuender, COVID-19-positiver Schwangerer gerechnet.

**Ziel der Arbeit:**

Diese Arbeit vermittelt einen Einblick in die SARS-CoV-2-Infektionszahlen im Rahmen der geburtshilflichen Anästhesie zu Beginn der Pandemie sowie während der zweiten Infektionswelle in Deutschland.

**Methoden:**

Über das COALA-Register (COVID-19 related Obstetric Anaesthesia Longitudinal Assessment-Registry) wurden sowohl von März bis Mai 2020 als auch von Oktober 2020 bis Februar 2021 in Deutschland und der Schweiz wöchentlich prospektiv Daten zu Verdachts- und bestätigten SARS-CoV-2-Fällen bei Schwangeren zum Zeitpunkt der Geburt erhoben. Betrachtet wurden die Verteilung dieser auf die Anzahl der Geburten, Zentren und Erhebungswochen sowie mütterliche Charakteristika und Krankheitsverläufe.

**Ergebnisse:**

Neun Zentren haben im Verlauf 44 SARS-CoV-2-positive Schwangere zum Zeitpunkt der Geburt bei 7167 Geburten (0,6 %) gemeldet (3 Fälle auf 2270 Geburten (0,4 %) und 41 Fälle auf 4897 Geburten (0,8 %)). Berichtet wurden 2 schwere COVID-19-Verläufe (*n* = 1 mit Todesfolge nach ECMO, *n* = 1 mit ECMO überlebt). Bei 28 (68 %) Patientinnen verlief die Infektion asymptomatisch. Ein Neugeborenes wurde im Verlauf positiv auf SARS-CoV‑2 getestet.

**Schlussfolgerung:**

Mithilfe des Registers konnte das Auftreten von Fällen zu Beginn der Pandemie zeitnah eingeschätzt werden. Es traten sporadisch Verdachtsfälle bzw. bestätigte Fälle auf. Aufgrund fehlender flächendeckender Testung muss aber von einer Dunkelziffer asymptomatischer Fälle ausgegangen werden. Während der zweiten Infektionswelle wurden 68 % asymptomatische Fälle gemeldet. Jedoch kann es bei jungen, gesunden Patientinnen ohne das Vorliegen typischer Risikofaktoren zu schwerwiegenden Verläufen kommen.

## Abstract (en)

**Background:**

In the current pandemic regarding the infection with the SARS-CoV-2-virus and COVID-19 as the disease, concerns about pregnant women, effects on childbirth and the health of the newborn remain high. Initially, due to the early manifestation of the disease in younger patients, high numbers of COVID-19 patients in women needing peripartum care were expected.

**Objective:**

This article aims to provide a general overview over the beginning of the pandemic as well as the second wave of infections in Germany and Switzerland, regarding SARS-CoV‑2 positive pregnant women hospitalized for childbirth. We therefore launched a registry to gain timely information over the dynamic situation during the SARS-CoV‑2 pandemic in Germany.

**Material and methods:**

As part of the COVID-19-related Obstetric Anesthesia Longitudinal Assessment (COALA) registry, centers reported weekly birth rates, numbers of suspected SARS-CoV‑2 cases, as well as the numbers of confirmed cases between 16 March and 3 May 2020. Data acquisition was continued from 18 October 2020 till 28 February 2021. The data were analyzed regarding distribution of SARS-CoV‑2 positive pregnant women hospitalized for childbirth between centers, calendar weeks and birth rates as well as maternal characteristics, course of disease and outcomes of SARS-CoV‑2 positive pregnant women.

**Results:**

A total of 9 German centers reported 2270 deliveries over 7 weeks during the first wave of infections including 3 SARS-CoV‑2 positive cases and 9 suspected cases. During the second survey period, 6 centers from Germany and Switzerland reported 41 positive cases out of 4897 deliveries. One woman presented with a severe and ultimately fatal course of the disease, while another one needed prolonged ECMO treatment. Of the women 28 presented with asymptomatic infections and 6 neonates were admitted to a neonatal intensive care unit for further treatment. There was one case of neonatal SARS-CoV‑2 infection.

**Conclusion:**

The number of pregnant women infected with SARS-CoV‑2 was at a very low level at the time of delivery, with only sporadic suspected or confirmed cases. Due to the lack of comprehensive testing in the first survey period, however, a certain number of asymptomatic cases are to be assumed. Of the cases 68% presented as asymptomatic or as mild courses of disease but the data showed that even in young healthy patients without the presence of typical risk factors, serious progression can occur. These outcomes should raise awareness for anesthesiologists, obstetricians, pediatricians and intensive care physicians to identify severe cases of COVID-19 in pregnant women during childbirth and to take the necessary precautions to ensure the best treatment of mother and neonate. The prospective acquisition of data allowed a timely assessment of the highly dynamic situation and gain knowledge regarding this vulnerable group of patients.

## Kurze Hinführung zum Thema

Im Rahmen der Pandemie des SARS-CoV-2-Virus erlangte das Patientenkollektiv der Schwangeren früh Aufmerksamkeit. So musste angesichts des geringen Wissens über dieses neuartige Virus mit einem potenziell erheblichen Aufkommen peripartal zu betreuender COVID-19-positiver Schwangerer gerechnet werden. Neben Anstrengungen, eine sichere Versorgung der betroffenen Patientinnen unter größtmöglichem Schutz von Nichtinfizierten sowie Krankenhauspersonal zu gewährleisten, wurde der wissenschaftliche Austausch ein wichtiger Bestandteil zur Bewältigung der Situation.

## Einleitung

### Hintergrund

Im Rahmen der aktuellen Pandemie des „severe acute respiratory syndrome coronavirus 2“ (SARS-CoV-2) erlangte das Patientenkollektiv der Schwangeren früh Aufmerksamkeit. Initial wurde angesichts von Krankheitsfällen auch bei jüngeren Patienten, mitunter ohne bedeutsame Vorerkrankungen, mit einem erheblichen Aufkommen peripartal zu betreuender, „Coronavirus-disease-2019“(COVID-19)-positiver Schwangerer gerechnet.

Infektionen mit SARS-CoV-2 und COVID-19-Erkrankungen in der Schwangerschaft sind bereits mehrfach beschrieben, wobei die ersten Beschreibungen bis Mitte des Jahres 2020 aus Wuhan, Provinz Hubei in China, vorlagen [1, 2]. Große Registerstudien aus den USA, England und Deutschland haben seitdem bereits Daten zu SARS-CoV-2-positiven Schwangeren veröffentlicht [3–6] jedoch sind die publizierten Daten hinsichtlich peripartaler Infektionen überraschend spärlich, was durch tatsächlich niedrige Fallzahlen, aber auch durch eine hohe Dunkelziffer und bislang fehlende prospektive Untersuchungen erklärt werden könnte. Breit angelegte (Register‑)Studien bezüglich peripartaler Infektionsfälle unter Berücksichtigung der europäischen Versorgungsstandards sind daher wünschenswert.

Dies umso mehr, als zunächst die Sorge bestand, schwangere Patientinnen könnten gehäuft und besonders schwer betroffen sein. Diese Annahme stützte sich auf die Beobachtung einer erhöhten Vulnerabilität von Schwangeren hinsichtlich der saisonalen Influenza [7], aber auch auf die in der Vergangenheit bei „severe acute respiratory syndrome“ (SARS) [8] und „Middle East respiratory syndrome“ (MERS) [9] beobachtete Fallzahlhäufung. In bisherigen Studien sind sowohl asymptomatische als auch schwere Verläufe beschrieben worden, obgleich die überwiegende Anzahl der Fallserien milde bis moderate Verläufe aufwiesen [1, 10, 11]. Dabei zeigten die Patientinnen ähnliche klinische Symptome wie nichtschwangere COVID-19-Patienten [11]. Spezifischere Symptome bzw. aus anderem Kontext bekannte pathognomonische klinische Zeichen sind bei Schwangeren bisher nicht besonders hervorgehoben worden [2].

Im Rahmen des zu erwartenden Patientenaufkommens wurden vielerorts erhebliche Anstrengungen auf organisatorischer und logistischer Ebene unternommen, um unter den krankenhaushygienischen Vorgaben perspektivisch eine sichere Versorgung der betroffenen Patientinnen unter größtmöglichem Schutz von Nichtinfizierten sowie Krankenhauspersonal zu gewährleisten. Mitunter geschah dies durch Eröffnung spezieller, von der üblichen Schwangerenversorgung separierter, Versorgungseinheiten („COVID-Geburtshilfe-Station“ bzw. „COVID-Kreißsaal“).

Um eine möglichst gute Datenbasis für weitergehende Entscheidungen im Kontext der geburtshilflichen Versorgung – unter besonderer Berücksichtigung der anästhesiologischen Betreuung – zu erstellen und klinisch-organisationale Entscheidungen der geburtsmedizinischen bzw. geburtshilflich-anästhesiologischen Betreuung zu unterstützen, wurde eine prospektive Datensammlung relevanter Routinedaten im Kontext der geburtsmedizinischen Versorgung konzipiert. Zugrunde liegende Ideen waren die frühzeitige Disseminierung erster Daten zu peripartalen Verläufen COVID-19-erkrankter Patientinnen und die Beschreibung von Krankheitsverläufen mit Blick auf die weitere notwendige Bedarfsplanung.

## Methodik

### COALA-Register

Bei der im Folgenden beschriebenen Analyse handelt es sich um eine multizentrische, prospektive Datenerhebung von SARS-CoV-2-Infektionen zum Zeitpunkt der Geburt sowie des peripartalen Verlaufs SARS-CoV-2-positiver Schwangerer unter besonderer Berücksichtigung des anästhesiologischen wie intensivmedizinischen Verlaufs, das nachfolgend mit dem Akronym „COALA-Register“ (COVID-19 related *O*bstetric *A*naesthesia *L*ongitudinal *A*ssessment-Registry) abgekürzt wird.

Eine prospektive erweiterte Datenerhebung, insbesondere auch bei besonderen Patientengruppen wie z. B. Schwangeren im Rahmen von COVID-19, war zu Beginn der Pandemiesituation durch die örtliche Ethikkommission bewilligt worden (Ethikvotum der Ethikkommission der Universität Würzburg vom 25.03.2020, Aktenzeichen 63/20-kr, Erstkontakt 20.03.2020). Da es sich bei den erhobenen Daten zudem um Routinedaten handelt, gilt im bayrischen Einzugsgebiet zudem Artikel 27 des Bayerischen Krankenhausgesetzes, welcher es Ärztinnen und Ärzten gestattet, Patientendaten u. a. zu Forschungszwecken zu verwenden [12].

Die Mitglieder des Arbeitskreises Geburtshilfliche Anästhesie der Deutschen Gesellschaft für Anästhesiologie und Intensivmedizin (DGAI) erhielten eine kurze Beschreibung zum geplanten Projekt per Mail-Verteiler mit der Bitte um Rückmeldung bei Interesse und Möglichkeit der Teilnahme. Zusätzlich wurden die Teilnehmer einer Expertengruppe mit geburtshilflichem Fokus via kommerziellem Messenger-Dienst kontaktiert, die mehrheitlich ebenfalls dem Arbeitskreis Geburtshilfliche Anästhesie der DGAI angehörten.

Den Kollegen, die ein Interesse an einer prospektiven Datenerhebung bekundeten, wurde das Anschreiben an die Würzburger Ethikkommission sowie das Ethikvotum (AZ 63/20-kr) zur Verfügung gestellt, um ergänzend, gerade bei fehlender Voraussetzung gemäß den jeweiligen Krankenhausgesetzen der betreffenden Bundesländer, ggf. zusätzliche Beratungen seitens der lokalen Ethikkommission einzuholen.

### Datenerhebung

Teilnehmende Zentren wurden gebeten, ab dem 16.03.2020 (Kalenderwoche [KW] 12) in eine für die Datenerfassung erstellte Excel-Tabelle wöchentlich die Anzahl der erfolgten Geburten insgesamt, Geburten mit COVID-19-Verdacht sowie Geburten mit bestätigter SARS-CoV-2-Infektion der Mutter zu erheben. Zur Generierung zusätzlicher Daten wurden die Zentren ab dem 19.10.2020 (KW 43) erneut gebeten, die entsprechenden Daten zu erheben.

Bei SARS-CoV-2-positiven Fällen wurden die in Tab. 1 und 2 aufgeführten Daten aus den Routinedaten des stationären Aufenthalts zu Mutter und Kind erhoben.

**Table 1:** 

Mutter	
*Allgemeines*	
–	Alter
	Aktive Raucherin vor/während Schwangerschaft
	Pulmonale Vorerkrankung
	Kardiale Vorerkrankung
	Immunsuppression
	Immunsuppression durch Medikamenteneinnahme
Zusätzliche infektiologische Informationen	Bakterielle Infektion
	Influenzanachweis
Geburtsmodus	Spontanpartus
	Sectio
Analgesie Spontanpartus	PDA
	I.v.-Opioid
	Lachgas
	Andere/keine Analgesie
Anästhesie für Sectio	Allgemeinanästhesie
	PDA
	Spinalanästhesie
	CSE
Geburt	Geburt vor vollendeter 37. SSW
	Falls ja: Angabe der SSW
	PPH (Sectio: < 1 l oder bei Spp > 500 ml)
	Uterotonikum intraop./postop. – Oxytocin – Carbetocin – Sulproston
Indikation zur Sectio (falls zutreffend)	Laborwerte (z. B. Leberwerte, Thrombozytopenie)
	Präeklampsie
	„Fetal distress“
	Z. n. Sectio
	PROM
Postpartale Analgesie	Ibuprofen
	PDA
	Paracetamol
*COVID-19*	
Anamnese	Exposition zu infizierter Person
	Unklare Exposition
	SSW bei SARS-CoV-2-Diagnose
Symptome und Auffälligkeiten in der Diagnostik	Komplikationen fetal (Fetal distress, andere)
	Fieber
	Myalgie
	Unwohlsein
	Husten
	Dyspnoe
	Heiserkeit
	Durchfall
	Brustschmerz
	Sauerstoffinhalation vor Geburt
	Leukozytose
	Lymphopenie
	CRP ↑^a^
	PCT ↑^a^
	ASAT (GPT)/ASAT (GOT) ↑^a^
	ASAT (GOT) ↑^a^
	ALAT (GPT) ↑^a^
	Thrombozytopenie^a^
	CT, Hinweis für Pneumonie
Therapie	Sauerstofftherapie vor/nach Partus
	Antivirale Therapie vor/nach Partus
	Antibiotikatherapie vor/nach Partus
	Lungenreifebehandlung
	Kortikosteroide vor/nach Partus
Postpartaler Verlauf	Intensivaufenthalt
	Beatmungspflichtigkeit
	Sauerstoffpflichtigkeit
	ECMO

*PDA* Periduralanästhesie, *CSE* „combined spinal epidural“, *SSW* Schwangerschaftswoche, *Spp* Spontanpartus, *SARS-CoV‑2* „severe acute respiratory syndrome coronavirus 2“, *PPH* peripartale Hämorrhagie, *PROM* frühzeitiger Blasensprung („premature rupture of membranes“), *CT* Computertomographie, *ECMO* extrakorporale Membranoxygenierung

^a^Bewertung der Laborwerte: Abweichungen gemäß den Referenzbereichen des lokalen Labors

**Table 2:** 

Neonat
Normales Geburtsgewicht
LBW
VLBW
ELBW
Neonatale Asphyxie
Neonatal Death
Nabelschnurblut-pH, arteriell < 7,25; venös < 7,2
BE Nabelschnurblut, arteriell < −10; venös < −10
„Fetal death/stillbirth“ (Todgeburt)
APGAR 1 min < 8; 5 min < 8; 10 min < 8
Beatmungspflichtigkeit
Intensivaufenthalt
COVID-19-Nachweis, postpartal; im Verlauf

*LBW* „low birth weight“ (< 2500 g), *VLBW* „very low birth weight“ (< 1500 g), *ELBW* „extremely low birth weight“ (< 1000 g), *BE* „base excess“, *COVID-19* „coronavirus disease 2019“

Die Auswahl der Parameter orientierte sich an dem von Chen et al. im März 2020 publizierten Review zu den klinischen Charakteristika und dem vertikalen Transmissionspotenzial der SARS-CoV-2-Infektion bei 9 schwangeren Patientinnen [2].

Diese Erhebung sollte nach stattgehabter Entbindung aus den während des stationären Aufenthalts erfassten Routinedaten durchgeführt werden. Die Daten jeder Patientin wurden anonymisiert an die Autoren übermittelt und in eine entsprechende Übersicht eingepflegt.

Die Datenerhebung und Analyse erfolgte mit dem Datenverarbeitungsprogramm Excel (Microsoft, Redmond, USA). Die Deskription der Daten erfolgt anhand von absoluten Zahlen sowie Verhältnissen (%) sowie von Mittelwerten mit Standardabweichung bzw. des Medians mit Interquartilen.

## Ergebnisse

### Allgemeine Registerdaten

25 Kliniken in Deutschland und der Schweiz äußerten Interesse an der Teilnahme an dem Register und wurden erneut kontaktiert. Hiervon meldeten sich 19 Zentren zurück und erhielten die oben beschriebenen Dokumente mit den in Tab. 1 und 2 aufgeführten Items zur Datenerfassung. Neun Kliniken erstatteten regelmäßig Bericht und übermittelten wöchentliche Geburtenzahlen; vier Kliniken meldeten in unregelmäßigem Abstand, ob Verdachtsfälle oder Fälle aufgetreten waren. Acht Kliniken beteiligten sich im Verlauf nicht an der weiteren Datenerhebung, wobei nachvollziehbare Argumente bezüglich Datenschutz sowie ein fehlendes, allgemeines Ethikvotum als Gründe aufgeführt wurden. Hinsichtlich der Versorgungsstufen der beteiligten Klinken liegen alle, bis auf ein Zentrum mit < 500 Betten, in den höheren Versorgungsstufen der jeweiligen Bundesländer (> 500 Betten).

Von Beginn der Datenakquise zum 16.03.2020 (KW 12) bis zum 03.05.2020 (Ende KW 18) liegen 7 Wochen prospektiv erhobener geburtshilflicher Daten vor. Insgesamt konnten in diesem Zeitraum 2270 Geburten aus 9 Zentren analysiert werden. Nicht jedes dieser Zentren übermittelte die wöchentlichen Geburtenzahlen. Pro Woche waren es im Median 372 Geburten. Jedes Zentrum verzeichnete in dem 7‑wöchigen Erhebungszeitraum im Median 252 Geburten.

Die zweite Datenerhebung im Rahmen einer zweiten Infektionswelle in Deutschland erfolgte über 19 Wochen, vom 19.10.2020 (KW 43) bis 28.02.2021 (KW 8). Es beteiligten sich 6 Zentren aus Deutschland und der Schweiz an der wöchentlichen Datenakquise und meldeten insgesamt 4897 Geburten.

### Geburten, Verdachtsfälle und bestätigte Fälle

Insgesamt wurden über den ersten genannten Zeitraum 3 Fälle einer bestätigten Infektion mit SARS-CoV‑2 gemeldet, was 0,1 % aller registrierten Geburten entspricht; neun klinische SARS-CoV-2-Verdachtsfälle (0,4 %) wurden gemeldet, die sich nicht bestätigt hatten. Eine Übersicht über die allgemeinen Zahlen liefert Tab. 3, und in Tab. 4 wird die Verteilung nach Zentren dargestellt.

**Table 3:** 

	Geburten	Verdachtsfälle	Bestätigte Fälle
*1. Erhebungszeitraum*			
n (%)	2270	9 (0,4)	3 (0,1)
Median (IQR^a^) pro KW^b^	372 (289;357)	1 (1;2)	0 (0;1)
Median pro Zentrum	252 (184;352)	1 (0;1)	0 (0;0)
*2. Erhebungszeitraum*			
n (%)	4897	43 (0,9)	41 (0,8)
Median (IQR) pro KW^a^	254 (234;296)	1 (0;5)	2 (1;3)
Median pro Zentrum	522 (537;960) s	1 (0;6)	3 (2;10)

^a^„Interquartile range“ (IQR)

^b^Kalenderwoche (KW)

**Table 4:** 

Zentrum	Anzahl der betrachteten Wochen	Geburten			Verdachtsfälle	Bestätigte Fälle	
		Gesamt	Anzahl im Mittel pro Woche (±SD)	Pro Jahr (Milupa-Liste 2019^b^)^a^	Pro Zentrum, *n* (%)	Pro Zentrum, *n* (%)	Gesamt (*n* = 3)
001	7	357	51 (6)	2500	2 (0,56)	1 (0,28)	1/3
002	6	458	76 (5)	3700	2 (0,44)	0 (0)	0
003	6	149	25 (6)	1600	0 (0)	0 (0)	0
004	7	348	50 (5)	3000	1 (0,29)	0 (0)	0
005	7	274	39 (5)	2400	0 (0)	0 (0)	0
006	7	220	31 (4)	1600	1 (0,45)	0 (0)	0
007	1	5	5 (0)	4000	1 (20)	0 (0)	0
008	7	229	33 (5)	2000	1 (0,44)	0 (0)	0
009	4	230	58 (4)	3300	1 (0,43)	2 (0,87)	2/3

^a^Angabe der Geburten pro Jahr näherungsweise, um die Anonymisierung der Zentren zu wahren

^b^[13]

In der zweiten Welle (19 Wochen) wurden insgesamt 41 bestätigte SARS-CoV-2-Infektionen bei 4897 Geburten an 6 Zentren gemeldet. Verdachtsfälle waren aufgrund von Routinetestungen retrospektiv schwer zu definieren.

Jedes Zentrum meldete im Median einen Verdachtsfall (Interquartilabstand [IQR] = 1). Hierbei handelte es sich um Patientinnen, die wegen aufgetretener Symptomatik, eines bestätigten oder berichteten Kontakts zu einer infizierten Person oder eines Aufenthalts in einem Risikogebiet abgestrichen oder gemäß zum Hospitalisationszeitraum gültiger lokaler Standards als mögliche SARS-CoV-2-Patientin behandelt wurden. Bei den als Verdachtsfällen klassifizierten Patientinnen (zumeist klinischer Verdacht bei Vorliegen COVID-typischer Symptomatik) war gleichwohl der erfolgende Abstrich (RT-PCR-Test sowie Testungen mittels Antigenschnelltest) negativ.

### Screeningverfahren

Im Verlauf der 7 Wochen kam es hinsichtlich routinemäßiger Abstriche zu Änderungen im Prozedere. Während in Kalenderwoche 12 das Routinescreening bei stationärer Aufnahme an nur einem Zentrum durchgeführt wurde, erfolgte dies in KW 14 bereits an 4 und in KW 18 an 5 teilnehmenden Zentren. Vier von 10 Zentren, die Rückmeldung über das angewandte Screeningverfahren gaben, führten auch bis Ende des ersten Erhebungszeitraums keine Routineabstriche aller Patientinnen durch.

Insgesamt liegt damit im analysierten Zeitraum bei 384 Patientinnen der insgesamt 2270 im Rahmen der Surveillance erfassten Patientinnen (17 %) ein negatives Testergebnis vor. Bei den übrigen Patientinnen wurde gemäß der lokalen Teststrategie nur dann ein Rachenabstrich durchgeführt, wenn ein hinreichender Verdacht auf eine SARS-CoV-2-Infektion vorlag. Hierzu zählten das Vorliegen COVID-19-typischer Symptome, Kontakt zu SARS-CoV-2-positiven Personen in der Anamnese, Aufenthalt in Risikogebieten (z. B. zu Beginn der Pandemie aufgrund der Reiseanamnese).

Alle Zentren, die in der zweiten Welle ihre Daten übermittelten, testeten ihre Patientinnen routinemäßig. Bei respiratorischen Symptomen oder bekanntem Kontakt zu COVID-19 kamen ggf. zusätzliche Schnelltests zum Einsatz.

### Bestätigte SARS-CoV-2-Fälle zum Zeitpunkt der Entbindung

Im Erhebungszeitraum der ersten Welle wurden insgesamt 3 SARS-CoV-2-positive Patientinnen (0,13 %) aus 2 Zentren identifiziert. An den restlichen beteiligten Kliniken war bis zum Ende der ersten Erhebung keine Schwangere zur Entbindung als SARS-CoV-2-positiv identifiziert worden.

In Erhebungszeitraum der zweiten Welle ließen sich 41 SARS-CoV-2-positive Patientinnen identifizieren (0,84 %). Jedes Zentrum meldete im Median 3 Fälle, wobei in 2 Zentren nur 2 Fälle aufgetreten waren, während ein Zentrum 18 Fälle zu verzeichnen hatte.

Wesentliche Information zu den in dieser Erhebung positiv getesteten Patientinnen finden sich in Tab. 5.

**Table 5:** 

COVID-19-Patientinnen	1. Erhebung *n* = 3	2. Erhebung *n* = 41	Gesamt *n* = 44
Alter der Patientinnen (Jahre)	35 (±7)	31 (±5)	32 (±5)
**Geburt**			
*Geburtsmodus*			
Sectio	1 (33)	9 (22)	10 (23)
Spontanpartus	2 (67)	32 (78)	34 (77)
*Anästhesie für Geburt*			
Allgemeinanästhesie	1 (33)	1 (2)	2 (5)
Spinalanästhesie	0	8 (20)	8 (18)
Periduralanästhesie	0	8 (20)	8 (18)
Weiteres (u. a. i.v.-Opioide)	0	15 (37)	15 (34)
*PPH*	*0*	*6 (15)*	*6 (14)*
*Uterotonikum intraop./postop.*	*1 (33)*	*34 (83)*	*35 (80)*
*Indikation zur Sectio (falls zutreffend)*	*n* = 1	*n* = 9	*n* = 10
Laborwerte (z. B. Leberwerte, Thrombozytopenie)	0	1 (11)	1 (10)
Präeklampsie	0	0	0
„Fetal distress“	0	2 (22)	2 (20)
Zustand nach Sectio	0	4 (44)	4 (40)
PROM	0	0	0
COVID-19	1 (100)	0	1 (10)
**Anamnese**			
*Aktive Raucherin*	*0*	*1 (2)*	*1 (2)*
*Bekannte Vorerkrankungen (pulmonal, kardial)*	*0*	*2 (5)*	*2 (5)*
*Erkrankungsbedingte Immunsuppression*	*0*	*1 (2)*	*1 (2)*
*Immunsuppression durch Medikamenteneinnahme*	*0*	*0*	*0*
*Exposition zu infizierter Person*	*3 (100)*	*4 (10)*	*7 (16)*
*Schwangerschaftswoche bei SARS-CoV-2-Diagnose*	*31(±0)*	*38(±2)*	*38(±3)*
*SARS-CoV-2-Diagnose postpartal*	2 (66)	0	2 (5)
**Symptome und Auffälligkeiten in der Diagnostik**			
*Komplikationen, fetal (Fetal distress, andere)*	*0*	*3 (7)*	*3 (7)*
*Keine Symptome*	*1 (33)*	*27 (66)*	*28 (64)*
*Symptomatische COVID-19-Erkrankung*	*2 (66)*	*14 (34)*	*16 (36)*
*Labordiagnostik*			
*Erhöhte Entzündungsparameter (Leukozyten, CRP, PCT)*	*2 (66)*	*24 (66)*	*26 (59)*
*Thrombozytopenie*^a^	*0*	*10 (24)*	*10 (23)*
*Erhöhte Leberwerte (GOT, GPT, GPT/GOT)*	*2 (66)*	*1 (2)*	*3 (7)*
*CT, Hinweis für Pneumonie*	*2 (66)*	*0*	*2 (5)*
*Sauerstoffinhalation vor Geburt*	*1 (33)*	*1 (2)*	*2 (5)*
**Therapie**			
*Sauerstofftherapie*	*2 (66)*	*1 (2)*	*3 (7)*
*Antivirale Therapie (prä-, postpartal)*	*0*	*0*	*0*
*Antibiotikatherapie*^b^	*2 (66)*	*10 (24)*	*12 (27)*
*Lungenreifebehandlung*	*0 (0)*	*1 (2)*	*1 (2)*
*Kortikosteroide*	*1 (33)*	*0 (0)*	*1 (2)*
**Verlauf postpartal**			
*Intensivaufenthalt*	*2 (66)*	*1 (2)*	*3 (7)*
*Beatmungspflichtigkeit*	*2 (66)*	*0 (0)*	*2 (5)*
*ECMO*	*2 (66)*	*0 (0)*	*2 (5)*
**Outcome**			
*Genesen*	*2 (66)*	*41 (100)*	*43 (98)*
*Verstorben*	*1 (33)*	*0 (0)*	*1 (2)*

*n *(%), Mittelwert (±Standardabweichung)

*SSW* Schwangerschaftswoche, *PPH* peripartale Hämorrhagie, *PROM* frühzeitiger Blasensprung („premature rupture of membranes“), *COVID-19* „coronavirus disease 2019“, *SARS-CoV‑2* „severe acute respiratory syndrome virus type 2“, *PCT* Prokalzitonin, *GOT* Aspartat-Aminotransferase, *GPT* Alanin-Aminotransferase, *CT* Computertomographie, *ECMO* extrakorporale Membranoxygenierung

^a^Bewertung der Laborwerte: Abweichungen gemäß lokalem Labor

^b^Antibiotikaprophylaxe bei Sectio caesarea ausgenommen

### Neonaten SARS-CoV-2-positiver Mütter

Eine Übersicht über die Neugeborenen beider Erhebungen finden sich in Tab. 6.

**Table 6:** 

Neugeborene	1. Erhebung *n* = 3	2. Erhebung *n* = 41	Gesamt *n* = 44
Frühgeburt	2 (66)	3 (7)	5 (11)
Normales Geburtsgewicht	1 (33)	38 (93)	39 (89)
LBW, VLBW, ELBW	1 (33)	3 (7)	4 (10)
Neonatale Asphyxie	0 (0)	2 (5)	2 (5)
„Neonatal death“	0 (0)	0 (0)	0
pH, Nabelschnur, Arterie < 7,25, Vene < 7,2	1 (33)	16 (39)	17 (39)
BE Nabelschnurblut arteriell <−10; venös <−10	0 (0)	22 (54)	22 (50)
„Fetal death/stillbirth“ (Todgeburt)	0 (0)	0 (0)	0
APGAR 1 min < 8; 5 min < 8; 10 min < 8	0 (0)	5 (12)	5 (11)
Beatmungspflichtigkeit (invasiv/nichtinvasiv)	1 (33)	3 (7)	4 (10)
Intensivaufenthalt	1 (33)	5 (12)	6 (14)
SARS-CoV-2-Nachweis postpartal/im Verlauf	0 (0)	1 (2)	1 (2)

*n *(%)

*n.b.* nicht bekannt, *LBW* „low birth weight“ (<2500 g), *VLBW* „very low birth weight“ (<1500 g), *ELBW* „extremely low birth weight“ (<1000 g), *BE* „base excess“, *COVID* „coronavirus disease“, *SARS-CoV‑2* „severe acute respiratory syndrome virus type 2“

## Diskussion

Das beschriebene Projekt wurde zu Beginn der Pandemiesituation in Deutschland ins Leben gerufen, um den Verlauf der Pandemie für das Patientenkollektiv der Geburtshilfe frühzeitig prospektiv zu analysieren und von Beginn der Pandemie an wichtige Daten zum weiteren Verständnis der COVID-19-Erkrankung in der Schwangerschaft und zur Geburt zu erheben. Zudem war die bestmögliche Vorbereitung aufgrund erster auftretender Fälle im Hinblick auf zu ergreifende medizinische, logistische sowie strukturelle Maßnahmen intendiert, und eine zeitnahe Informationsweitergabe zu den Erkenntnissen aus diesen Daten via Messenger-Dienst bzw. E‑Mail-Verteiler des Arbeitskreises Geburtshilfliche Anästhesie.

Bei Interpretation der vorliegenden Daten sowie retrospektiv, in Kenntnis des Verlaufs der Pandemie, wird ersichtlich, dass die Lage im Hinblick auf an COVID-19 erkrankte Schwangere in Deutschland erfreulicherweise überschaubar geblieben ist. Auch wenn von einer gewissen Dunkelziffer nichtdetektierter asymptomatischer Fälle ausgegangen werden muss (kein flächendeckendes Screening mittels RT-PCR oder Antigenschnelltests zu Beginn der Pandemie), scheint sich die Zahl der mit SARS-CoV‑2 infizierten Schwangeren zum Zeitpunkt der Geburt auf niedrigem Niveau gehalten zu haben. An den beteiligten Zentren traten lediglich sporadisch klinische Verdachtsfälle bzw. bestätigte Fälle auf.

Eine sichere Aussage bezüglich negativem SARS-CoV-2-Status aller als negativ gemeldeter Patientinnen wäre nur dann möglich, wenn die Testung aller Schwangeren zur Geburt unabhängig von Symptomstatus oder Reisestatus durchgeführt wird. Da ein flächendeckendes Screening in den Kliniken zu unterschiedlichen Zeitpunkten umgesetzt wurde, lag nur bei einem Teil des Kollektivs ein eindeutiger Befund zum SARS-CoV‑2 Status vor. Klinik und Anamnese waren zu diesem Zeitpunkt, nicht nur allein aufgrund von fehlender Testkapazitäten, essenziell in der primären Diagnostik. An den meisten an dieser Untersuchung teilnehmenden Universitätskliniken mit hohen Geburtenzahlen und großem Einzugsgebiet hat es bis zum Ende der ersten Erhebung, welcher der primären Einschätzung der Pandemiesituation in unserem Patientenkollektiv zugrunde lag, keinen bestätigten Fall gegeben. Im Verlauf der Pandemie verzeichneten jedoch alle beteiligten Zentren positive SARS-CoV-2-Fälle bei Schwangeren zum Zeitpunkt der Geburt. Auch die Teststrategie hat sich im Einklang mit anderen Erhebungen zum Positiven – im Sinne einer liberaleren Routinetestung – gewandelt [14].

### Kommunikation während der Pandemiesituation

Durch das Projekt wurden kurzfristig relevante Daten in verschiedenen Kliniken erhoben und zentral gesammelt. Insbesondere in dieser zunächst von großen Unsicherheiten geprägten Pandemiesituation schien ein Austausch unter den Kliniken bedeutsam, um Trends und Tendenzen frühzeitig zu erkennen. Durch die anästhesiologischen Vertreter vor Ort wurde zudem das Bewusstsein für den innerklinischen Verlauf geschärft. Durch den wöchentlichen Austausch untereinander wären bei Auftreten hoher Fallzahlen eine frühzeitige Kommunikation und Dissemination zur Erarbeitung sinnvoller Vorkehrungen möglich gewesen. Darüber hinaus hätten bei Auftreten einer hohen Anzahl von COVID-19-Fällen zeitnah relevante Befunde, Krankheitsverläufe, klinische Merkmale und Behandlungsstrategien identifiziert und für einen zeitnahen Erkenntnisgewinn zur Verfügung gestellt werden können. So wurden an ähnlichen Kohorten klinische Überschneidungen zu Symptomen der Präeklampsie beschrieben [15] bzw. die Triggerung einer Immunthrombozytopenie aufgrund von Fallberichten zur Diskussion gestellt [16].

Aufgrund der Kurzfristigkeit der Erstellung dieses Registers, zumal während einer kapazitätenbindenden Pandemie, meldete sich lediglich eine überschaubare Anzahl an Vertretern aus wenigen Klinken. Zudem kam nicht durchgehend der intendierte regelmäßige Austausch zustande. Des Weiteren sind im Nachhinein Kliniken aus Landkreisen mit im Zeitraum der Erhebung hohen SARS-CoV-2-Fallzahlen, entsprechend den täglichen Lageberichten des RKI, nicht vertreten [17], sodass grundsätzlich eine gewisse Unterschätzung bei den getätigten Schlussfolgerungen nicht auszuschließen ist. Wünschenswert wäre zudem eine etablierte Netzwerk- bzw. Datenbankstruktur gewesen, sowie ein bereits ausgereifteres Datenschutzkonzept und situativ zu ergänzende Ethikvoten, die möglicherweise in einer breiteren Beteiligung gemündet hätten.

### Rückschlüsse möglich?

Durch die mutmaßlich niedrige Inzidenz von Infektionen mit SARS-CoV‑2 bei Schwangeren zur Geburt und die Heterogenität der berichteten Fälle werden die Ergebnisse an dieser Stelle rein deskriptiv dargestellt. Mithin sind Rückschlüsse auf das gesamte Patientenkollektiv in Deutschland oder die weitere Situation aufgrund der genannten Limitationen der vorliegenden Erhebung nur eingeschränkt möglich.

Auch durch die regional sehr variablen Infektionszahlen, sowohl in den einzelnen Städten als auch der jeweiligen Einzugsgebiete, haben Hochrechnungen im Hinblick auf Inzidenzen auf Basis der vorliegenden Zahlen eher spekulativen Charakter. In ganz Deutschland nahmen die SARS-CoV-2-Fälle im Zeitraum dieser Erhebung von zu Beginn 6,5 Fällen auf 100.000 Einwohner am ersten Tag der Erhebung bis 195 Fälle auf 100.000 Einwohner zu [18]. Zudem handelte es sich hierbei primär um eine Momentaufnahme aus dem Zeitraum Mitte März bis Anfang Mai 2020, die keine Aussagen über den längerfristigen Verlauf zulässt. Sekundär entschlossen sich die Autoren – angeregt nicht zuletzt durch das Peer-Review-Verfahren dieses Manuskriptes – Verlaufsdaten der zweiten Welle abzuwarten, um diese Arbeit auch inhaltlich mit Informationen zu den aufgetretenen Fällen in Deutschland zu bereichern. Einschränkend ist ferner zu berücksichtigen, dass in der vorliegenden Erhebung nur der Status zum Zeitpunkt der Geburt abgebildet wurde. Unterschiede zu Patientenkohorten mit SARS-CoV-2-Infektionen während der Schwangerschaft [19] sind zu erwarten.

Hinsichtlich des Krankheitsverlaufs bei Schwangeren führen insbesondere die 3 Fälle der ersten Welle eindrücklich vor Augen, dass eine allgemeine Aussage bzgl. der Erkrankungsschwere bei Schwangeren nicht möglich ist. SARS-CoV-2-Infektionen bei schwangeren Patientinnen können auch ohne Vorliegen der klassischen Risikofaktoren (hohes Alter, pulmonale oder kardiale Vorerkrankungen) schwerste Verläufe nehmen. Die Daten der zweiten Erhebung zeigen jedoch auch, dass eine Infektion mit SARS-CoV‑2 zum Zeitpunkt der Geburt asymptomatisch oder nur mild verlaufen kann (in dieser Kohorte 95 %).

Es zeigte sich ferner, dass eine wissenschaftliche rasche multizentrische Aufarbeitung aufgrund des hohen öffentlichen Interesses und individueller Sorgen bzw. gar eines Widerspruchs bezüglich der Datenanalyse sowie aufgrund der komplexen pandemischen Gesamtsituation erschwert war.

Entsprechend den allgemeinen Infektionszahlen der zweiten Welle in Deutschland, welche mit 440 Fällen auf 100.000 Einwohner am ersten Erhebungstag und 2937 Fällen auf 100.000 Einwohner am letzten [18] deutlich höher liegen als im Zeitraum der ersten Erhebung, passt auch die deutlich höhere Anzahl SARS-CoV-2-positiver Schwangerer zum Zeitpunkt der Geburt dieses Erhebungszeitraums [20]. Jedoch mit 0,84 % der gemeldeten Geburten ist weiterhin keine erhebliche Häufung erkennbar. Insgesamt präsentiert sich ein Bild vieler asymptomatischer und milder Verläufe mit einigen Zufallsbefunden im Rahmen des Screenings in der Klinik. Trotz alledem sollten, aufgrund der aus vorherigen Publikationen bekannten erhöhten Morbidität und Mortalität bei COVID-19 im Rahmen der Schwangerschaft [4, 21], SARS-CoV-2-positive Schwangere engmaschig überwacht werden.

Die Neugeborenen scheinen postpartal gesundheitlich nicht unmittelbar von der Infektion der Mutter beeinträchtigt zu sein. Es ist hervorzuheben, dass bei verschiedenen Krankheitsverläufen alle Neugeborenen überlebt haben. Neonatale Asphyxie und reduzierte APGAR-Werte, die mit einer Infektion der Mutter im Zusammenhang stehen können, wurden beschrieben. Die Frühgeburtlichkeitsrate in dieser Kohorte ist mit 12 % ähnlich der in vorherigen Studien zu COVID-19 in der Schwangerschaft beschriebenen (Allotoy et al., 12,4 %, Woodworth et al. 11,4 %) [21, 22]. Diaplazentare SARS-CoV-2-Übertragungen sind selten und werden in der Literatur mit 3–8 % der Geburten der während der Schwangerschaft mit SARS-CoV‑2 infizierten Müttern beschrieben. Im Nabelschnurblut einiger Neonaten gefundene IgM-Antikörper weisen zusätzlich darauf hin, dass Infektionen in utero nicht auszuschließen sind [23]. In dieser Kohorte wurde ein Neugeborenes im Verlauf positiv auf SARS-CoV‑2 getestet. Hierbei ist jedoch eine postnatale Infektion bei zusätzlichem Rooming-in nicht auszuschließen.

Eine mögliche Hypothese, für die geringe Infektionsrate zum Zeitpunkt der Geburt trotz Routinetestungen wäre, dass sich Schwangere aufgrund der Gesamtsituation und aus Sorge vor gesundheitlichen Risiken häufiger und u. U. auch deutlich frühzeitiger in Isolation begeben haben als die Normalbevölkerung. Dies muss gesondert betrachtet werden. Sicher ist, dass durch Vorgaben des Mutterschutzgesetzes und mitunter eines generellen Beschäftigungsverbotes, z. B. für patientennahe Tätigkeiten in der Krankenversorgung, eine deutliche Reduktion der Arbeitskontakte stattgefunden hat [24]. Vorstellbar wäre aber auch ein immunologischer Erklärungsansatz, etwa durch eine selektiv gestärkte Immunkompetenz, die Schwangere vor bestimmten Virusinfektionen schützen könnte [25], oder ein erhöhter Plasmaspiegel des „angiotensin-converting enzyme 2“, das zirkulierend eine wichtige Rolle bei der Abwehr der COVID-19-Infektion zu spielen scheint [26]. Allerdings ist bei anderen Virusinfektionen wie beispielsweise der Influenza gezeigt worden, dass gerade Schwangere empfänglicher für eine Infektion sind [27, 28]. Auch die veränderten Lungenvolumina sowie die hormonell bedingte Schwellung der Atemwege in der Schwangerschaft sprechen zunächst für ein höheres Risiko bzw. für gravierendere Auswirkungen einer Infektion [29].

### Zusammenfassung

Die Pandemie zeigte eindrücklich auf, dass eine rasche Verfügbarkeit von Daten im Hinblick auf die Disseminierung von Erkenntnissen zu Krankheitsverläufen wie auch Behandlungen ausgesprochen wünschenswert ist. Der beschriebene Ansatz einer frühzeitigen prospektiven Erhebung zu einem bestimmten Krankheitsbild ist zwar in Hinblick auf Datengenauigkeit weitaus weniger sicher und überprüfbar als auf dezidierte Case Report Forms basierende Register- bzw. Mortalitätsdaten [30, 31], könnte sich aber als Kompromiss bezüglich Aufwand und Ergebnis zur möglichst sicheren Erfassung von Inzidenzen von (seltenen) Komplikationen und (seltenen) Krankheitsverläufen herausstellen.

Aus Sicht der Autoren wäre es wünschenswert, den begangenen Weg einer selektiven Datenerhebung krankheits- bzw. komplikationsspezifisch weiterzuverfolgen, ohne den zweifelsohne fundierteren, aber aufwendigeren und weniger selektiven Weg einer umfassenderen Registererhebung [32] deshalb außer Acht zu lassen.

## Fazit für die Praxis

- Infizierte Gebärende traten an den teilnehmenden Kliniken nur sporadisch auf; Infektionszahlen SARS-CoV-2-positiver peripartal betreuter Schwangerer in Deutschland haben sich auf niedrigem Niveau gehalten.
- Bei schwangeren Patientinnen kann COVID-19 auch ohne Vorliegen der klassischen Risikofaktoren (hohes Alter, pulmonale oder kardiale Vorerkrankungen) schwerste Verläufe nehmen.
- Eine versierte pädiatrische Versorgung der Neugeborenen sollte, neben einer Expertise in der intensivmedizinischen Betreuung bei SARS-CoV-2-Infektion der Mutter, zum Zeitpunkt der Geburt gesichert sein.
- In einer von großen Unsicherheiten geprägten Pandemiesituation ist ein Austausch unter den Kliniken bedeutsam, um Trends und Tendenzen frühzeitig zu erkennen.
